# An Aggregation-defective Mutant of *Methanothermobacter* sp. CaT2 Reveals Unique Protein-dependent Aggregation

**DOI:** 10.1264/jsme2.ME19014

**Published:** 2019-06-13

**Authors:** Kana Sumikawa, Tomoyuki Kosaka, Noriaki Mayahara, Minenosuke Matsutani, Koichi Udo, Mamoru Yamada

**Affiliations:** 1 Applied Molecular Bioscience, Graduates School of Medicine, Yamaguchi University Yamaguchi 753–8515 Japan; 2 Department of Biological Chemistry, College of Agriculture, Graduates School of Science and Technology for Innovation, Yamaguchi University Yamaguchi 753–8515 Japan; 3 Research Center for Thermotolerant Microbial Resources, Yamaguchi University Yamaguchi 753–8515 Japan; 4 Science Research Center, Yamaguchi University Ube 755–8505 Japan

**Keywords:** hydrogenotrophic methanogen, aggregation, aggregation-defective mutant, genomic analysis, adhesion protein

## Abstract

The thermophilic hydrogenotrophic methanogen, *Methanothermobacter* sp. CaT2, which possesses an extracellular sugar layer, commonly aggregates by itself or with other microorganisms. To elucidate the molecular mechanisms responsible for this aggregation, the aggregation-defective mutant, CLA160, was isolated. Optical and electron microscopy observations revealed that the mutant exhibited a significant reduction in aggregation. Genomic sequencing showed that CLA160 has a single point mutation, causing a nonsense mutation in *MTCT_1020*, which encodes a hypothetical protein. Motif and domain analyses indicated that the hypothetical protein bears two membrane-spanning segments at the N- and C-terminal regions and a large middle repeat-containing region. The results of a bioinformatic analysis suggested that the first middle region (RII) of the protein or the whole structure is responsible for the function of the product of *MTCT_1020* in the aggregation of CaT2. A treatment with proteinase K suppressed sedimentation in CaT2, indicating a reduction in aggregation, with almost no effect on sedimentation in CLA160. The addition of Ca^2+^ or Mg^2+^ ions enhanced sedimentation in CaT2, whereas a DNase treatment had no effect on sedimentation in either strain. These results suggest that the hypothetical protein encoded by *MTCT_1020* plays a key role as a membrane-bound adhesion protein in the aggregation of CaT2, which is enhanced by the addition of Ca^2+^ or Mg^2+^ ions.

Methane fermentation is performed under mesophilic (approximately 37°C) or thermophilic (approximately 55°C) conditions in bioreactors, among which the upflow anaerobic sludge blanket (UASB) reactor is the most widely and effectively used (32, 38). Efficient fermentation in UASB-type reactors depends on the formation and activity of granules (19, 33). Granules are composed of many types of microorganisms, including primary fermenting bacteria, secondary fermenting bacteria, acetoclastic methanogens, and hydrogenotrophic methanogens (18, 30, 33, 34). Methanogens are considered to be key species in granule formation (7, 38, 44). Methanogens in the granules that form in thermophilic reactors produce two- to three-fold more methane than methanogens in granules in mesophilic reactors (11, 25). However, granule formation is generally more difficult in thermophilic reactors than in mesophilic reactors (11). This phenomenon may be attributed to stability differences in aggregation mechanisms or responsible molecules under mesophilic and thermophilic conditions. Therefore, a clearer understanding of the aggregation mechanisms of thermophilic methanogens is important when considering the stabilization of UASB reactors.

Cellular aggregation has been reported in many microorganisms; it is accomplished via aggregating substances, such as filaments, extracellular DNA (eDNA), proteins, lipids, and polysaccharides (10, 19, 20, 25, 39). Several aggregating methanogens, particularly mesophilic methanogens, have been reported to date, and their aggregation substances and mechanisms have been investigated. *Methanosarcina mazei* produces polysaccharide aggregates and forms extracellular polymers (ECPs) that attach to the outer surfaces of cells (27). *Methanobacterium formicicum* and *Methanobrevibacter* sp. produce ECPs to bridge the gap between aggregates formed by other species (33, 39). *Methanococcus maripaludis* uses flagella and pili to adhere to the surfaces of other microbes (12). Therefore, mesophilic methanogen aggregation mechanisms using polysaccharides, ECPs, and proteins have been proposed. However, research on aggregating thermophilic methanogens is limited. The thermophilic aggregating hydrogenotrophic methanogen, *Methanothermobacter* sp. CaT2, was recently reported (16). A physiological analysis of CaT2 suggested that its cell-surface sugar layer is responsible for its aggregation properties (16). The genome of this self-aggregating thermophilic methanogen has been sequenced and its genetic background revealed (15). A comparative genomic analysis with other thermophilic hydrogenotrophic methanogens indicated that CaT2 contains specific aggregationrelated adhesion genes and suggested that pili (fimbriae) do not affect strong cell-to-cell aggregation of CaT2 (16). However, no clear relationship between the physiology and genetics of CaT2 has been identified, and its aggregation mechanisms currently remain unclear.

In order to elucidate the molecular mechanisms underlying the cell-to-cell aggregation of CaT2 in more detail, we isolated the mutant, CLA160, which displayed significantly decreased aggregation. A mutation analysis revealed that CLA160 has a nonsense mutation in a gene for a hypothetical membrane protein with a large extracellular domain. In addition, a proteinase K treatment suggested that proteins on the membrane or cell wall are responsible for aggregation in CaT2.

## Materials and Methods

### Strains and growth conditions

*Methanothermobacter* sp. CaT2 (DSM 24414 and NBRC 107770) and its mutant derivatives were used in the present study. Strains were grown in W medium at 55°C as previously described (16), or W-R medium (W medium without resazurin solution) under a controlled atmosphere: 160 kPa H_2_/CO_2_ (80/20 [v/v]) (Sumitomoseiki, Osaka, Japan).

The aggregation-defective mutant CLA160 was isolated as follows. To increase mutation frequency, ethyl methanesulfonate (EMS) was used as a mutagen. The EMS treatment was performed according to a previously reported method (42). EMS was added to a culture of CaT2 at a final concentration of 0.5 M and cells were incubated at 37°C. Cells in the culture were collected by centrifugation and washed once with a 6% w/v sodium thiosulfate solution, washed twice with dH_2_O, and inoculated into fresh W-R medium under H_2_/CO_2_ (80/20 [v/v]). Mutagen-treated cells were passed through a 5-μm filter to remove aggregated cells and the cell suspension was then centrifuged. The pellet was suspended in 1 mL of dH_2_O and 100 μL of the suspension was mixed in W medium gellan gum plates for colonies to grow following a previously described method (24). Each colony was then grown in W medium under H_2_/CO_2_ (80/20 [v/v]) and aggregation was examined several times using a phase-contrast microscope (Eclipse E600; Nikon, Tokyo, Japan) to check the characteristics of the mutants.

### Phase-contrast, scanning electron microscope (SEM), and transmission electron microscope (TEM) images

A fluorescence microscope (Eclipse E600; Nikon) was used to take phase-contrast images. SEM images were taken using the following procedure: Cell pellets were fixed for 3 h with 1.25% glutaraldehyde in 0.1 M cacodylate buffer (pH 6.5) at 4°C, washed with 0.1 M cacodylate buffer, and then suspended with a small amount of the same buffer. The suspension was dropped on a 0.2-μm cellulose membrane filter and dehydrated with a graded ethanol series and t-butyl alcohol. The dehydrated filter was dried using vacuum freeze-drying equipment (VFD-21S; Vacuum Device, Ibaraki, Japan). The dried membrane filter was then coated with platinum (Auto Fine Coater, JFC-1600; JEOL, Tokyo, Japan). Images of sections were obtained by observations using SEM (10 kV, 30 μs; JSM-6360LA; JEOL). TEM with ruthenium red was performed as previously described (16), with slight modifications. Cell pellets were fixed for 5 h with 1.25% glutaraldehyde and 0.5 mg mL^−1^ ruthenium red in 0.1 M cacodylate buffer (pH 6.5) at 4°C, washed in 0.1 M cacodylate buffer, and post-fixed for 2 h in 1% osmium tetroxide containing 0.5 mg mL^−1^ ruthenium red in 0.1 M cacodylate buffer at 4°C. After being rinsed with 0.1 M cacodylate buffer, the pellets were embedded in a 3% agarose gel and dehydrated with a graded ethanol and acetone series. The dehydrated blocks were embedded in Epok 812 (Kohken, Tokyo, Japan). Ultra-thin sections were cut using a diamond knife mounted in an ultramicrotome (EM-UC6; Leica, Wetzlar, Germany), then placed on copper grids and stained with uranyl acetate and lead citrate. TEM examinations were performed using a Tecnai G2 Spirit electron microscope (Thermo Fisher Scientific, Massachusetts, USA) at an acceleration voltage of 80 kV.

### Gas-phase analysis

Methane and hydrogen were analyzed using a gas chromatography system (GC-8A and C-R6A; Shimadzu, Kyoto, Japan) with a thermal conductivity detector and column (2 m×3 mm stainless steel) packed with Unibeads C 60–80 (GL Science, Tokyo, Japan). Analysis conditions were as follows: injection at 150°C, column at 145°C, detector at 150°C, current for the detector at 50 mA, and flow rate of carrier Ar gas at 30 mL min^−1^.

### Sedimentation index assay with various treatments

Cells of strains were grown in W-R medium at 55°C for 4 d under H_2_/CO_2_ (80/20 [v/v]), recovered by centrifugation, washed twice, and suspended in 50 mM Tris-HCl buffer (pH 7). Suspensions (OD_600_ *ca*. 0.7–1.5) were subjected to optical density measurements or to the following treatments. In the proteinase K treatment, proteinase K (Wako Pure Chemical Industries, Osaka, Japan) was added to the cell suspension at a final concentration of 10 μg mL^−1^ and the solution was incubated at 37°C for 1 h. The control examination was performed by the addition of 50 mM Tris-HCl buffer instead of proteinase K. After the incubation, cells were washed once with 1 mL of 50 mM Tris-HCl buffer and subjected to a sedimentation index assay. Regarding the DNase I, CaCl_2_, and MgCl_2_ treatments, DNase I, CaCl_2_, MgCl_2_, or Tris-HCl buffer was added to the cell suspension at a final concentration of 5 μg mL^−1^, 10 mM, 10 mM, and 50 mM, respectively, and solutions were incubated at 37°C for 1 h. The control examination was performed by adding 50 mM Tris-HCl buffer instead of DNase I, CaCl_2_, or MgCl_2_. After the incubation, cells were washed once and suspended in 50 mM Tris-HCl buffer. To compare the speed of the sedimentation of cells, these cell suspensions were mixed briefly and their turbidity was measured at OD_600_ every 10 mins for 50 min using a photometer (Libra S12; Berthold Technologies, Bad Wildbad, Germany). The sedimentation index was calculated using the following equation: Sedimentation index=(the value of turbidity at each measured time)/(the value of turbidity at 0 min)×100.

### Extraction of genomic DNA

Cells were grown in 500 mL of W medium at 55°C for 4 d under H_2_/CO_2_ (80/20 [v/v]), recovered by centrifugation (12,000 rpm) at 4°C for 15 min, and washed twice with dH_2_O. Cell pellets were suspended in TE buffer. The suspension was then subjected 5 times to a freeze-thaw treatment of freezing at −80°C for 30 min and thawing at 55°C for 10 min. The treated solution was incubated overnight at 55°C following the addition of SDS, proteinase K, NaCl, and RNase A, at final concentrations of 0.5% (w/v), 100 μg mL^−1^, 10 mM, and 10 μg mL^−1^, respectively. After the incubation, the solution was gently mixed by inversion with double the volume of TE-saturated phenol. A half-volume of chloroform was added, and the solution was kept on ice for 5 min before being centrifuged at 14,000 rpm for 15 min at 4°C. Sodium acetate (pH 8) was then added to the transferred upper aqueous layer to a final concentration of 0.3 M. The solution was gently mixed with an equal volume of isopropanol and centrifuged at 14,000 rpm for 15 min at 4°C. The precipitate was washed with an equal volume of 70% (v/v) ethanol and centrifuged at 14,000 rpm for 15 min at 4°C. The resultant precipitate was dried and resuspended in 100 μL of TE buffer. The sample of genomic DNA was further purified using a Genomic-tip 20 kit (Qiagen, Hilden, Germany), according to the manufacturer’s instructions.

### Genome sequencing and mapping analysis of CLA160 strains against CaT2 genome sequences

We previously reported the complete genome sequence for the *Methanothermobacter* sp. CaT2, which is available at DDBJ/EMBL/GenBank, accession numbers AP011952.1 and AP011953.1 (15). To identify the mutation sites of CLA160, we performed genome sequencing of CLA160 using the Illumina Hiseq 2000 platform and mapped this against the complete genome sequence of CaT2. The genome sequencing of CLA160 was performed as previously reported (21). A total of 6,207,761 sequence pairs of 100 bp paired-end nucleotide reads from CLA160 were obtained, which yielded approximately 717-fold sequence coverage. The Illumina sequencing reads of CLA160 were aligned with the CaT2 genome sequence using BWA (17). Mutation sites were searched for using the Genome Analysis Toolkit (GATK) v 2.1.8 (22). Predicted mutation sites were analyzed by direct sequencing using appropriate primers: for 1022836, CLAm02_F: GAATATGGGTCTCGCCGTTA, CLAm02_R: AGG TAATGGCACCATTTCAGG; for 1180340, CLAm13_F: CCGA TACAGAGAAGACCCTCC, CLAm13_R: CAGCATATTACTTG AGGCGACAG; and for 1614615, CLAm14_F: CACAAGTGCCTTCATGGTTAC, CLAm14_R: TGTCTCATGCACACATCACC. Direct sequencing was performed using an ABI 3130XL sequencer (Applied Biosystems, California, USA) with a Big Dye Terminator v3.1 Cycle Sequencing Kit (Applied Biosystems), according to the manufacturer’s instructions.

### Sequence data deposition

Illumina sequence reads of the CLA160 strain have been deposited in the DDBJ Sequence Read Archive under the accession number DRA006429. The BioProject ID of the CAL160 strain is PRJDB6667.

### Bioinformatics analyses

An HMM model search was performed by hmmsearch using the HMMER web server (https://www.ebi.ac.uk/Tools/hmmer/search/hmmsearch) (8, 9). DELTA-BLAST was performed using the NCBI web server (https://blast.ncbi.nlm.nih.gov/Blast.cgi). The selection of similar proteins from the DELTA-BLAST result was performed using a ruby script under the condition: E-value <1.0E–10, sequence coverage >85%, identity >20%.

## Results

### Characterization of the aggregating strain CaT2 and aggregation-defective mutant CLA160

**Fig. 1:**
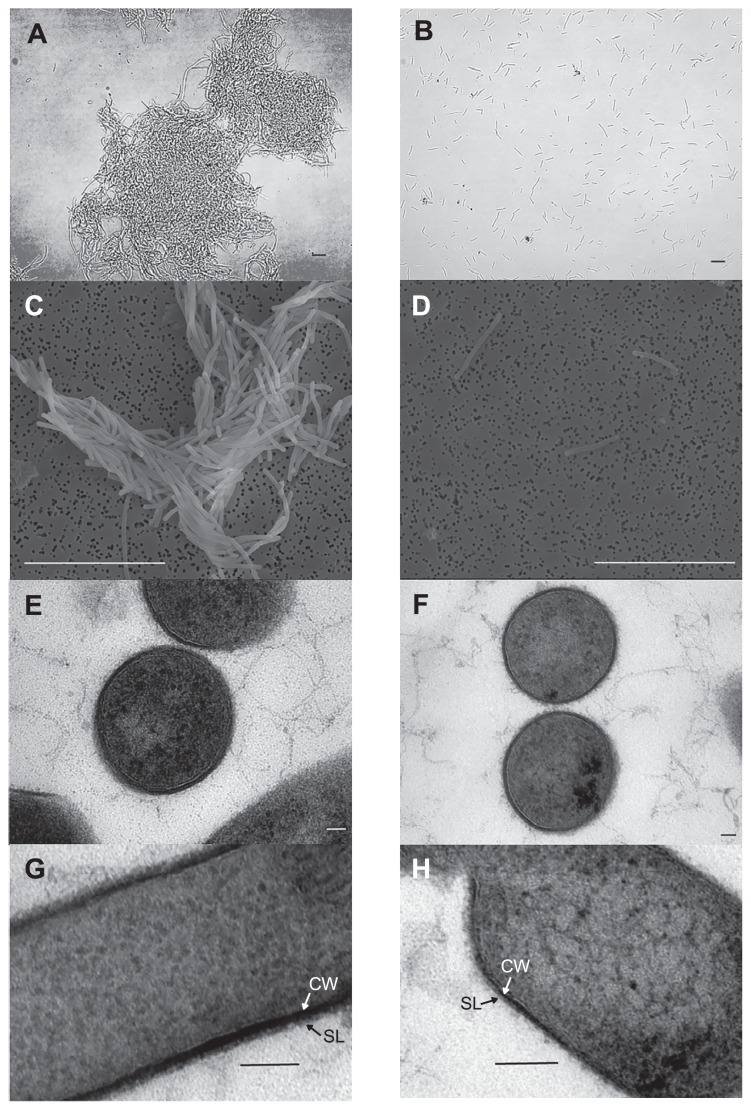
Images of *Methanothermobacter* sp. CaT2 and the aggregation-defective mutant CLA160. Phase-contrast images of CaT2 (A) and CLA160 (B) taken at ×400 magnification. SEM images of CaT2 (C) and CLA160 (D) were taken at ×12,000 magnification. TEM images of CaT2 (E, G) were taken at ×68,800 and ×430,000 magnification, respectively, and those of CLA160 (F, H) were taken at ×60,200 and ×430,000 magnification, respectively. SL, sugar layer; CW, cell wall. Bars represent 10 μm in A, B, C, and D; 50 nm in E and F; and 100 nm in G and H.

**Fig. 2:**
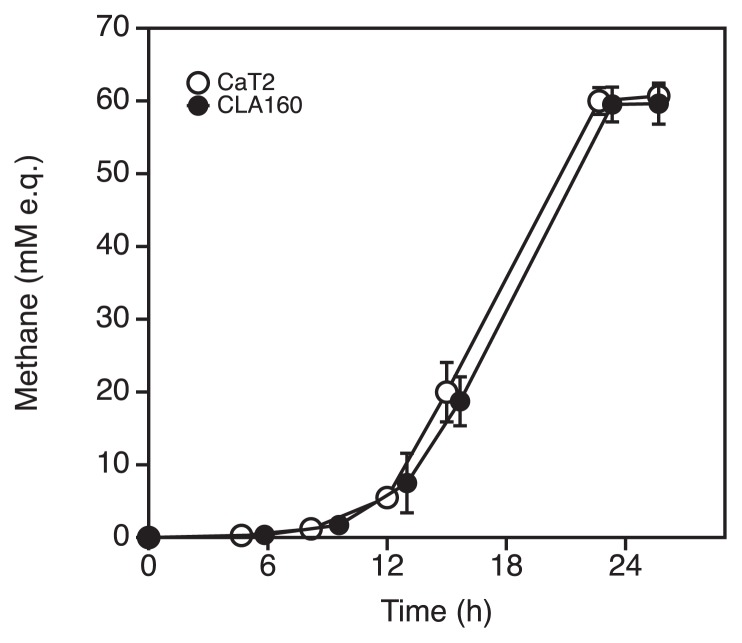
Comparison of methane production by CaT2 and CLA160. CaT2 (open circles) and the aggregation-defective mutant CLA160 (closed circles) were grown in W medium under H_2_/CO_2_ (80/20 [v/v]) and methane production was measured. Error bars represent standard deviations of the means of two independent experiments.

*Methanothermobacter* sp. CaT2 cells exhibited strong aggregation in W medium. The sugar layer on the cell surface of CaT2, which was stained by ruthenium red, was assumed to contribute to the aggregation of CaT2 (16). To elucidate the mechanisms underlying CaT2 aggregation, the aggregation-defective mutant, CLA160, was obtained. Since there are no methods for gene engineering, including gene disruption, in *Methanothermobacter* sp., an EMS treatment was applied for the acquisition of aggregation-defective mutants. Following cultivation, one of these mutants, CLA160, was compared with the parental strain, CaT2, using microscopy. Phase-contrast images indicated that CaT2 and CLA160 showed significant aggregation and almost no aggregation, respectively (Fig. 1A and B). SEM observations revealed that individual cells of CaT2 bound with other cells via interactions at their long axes, whereas CLA160 cells showed no such interactions (Fig. 1C and D). In addition, SEM revealed that CaT2 and CLA160 both exhibited similar smooth cell-surface structures (Fig. 1C and D). Moreover, sugar staining observations indicated that the sugar layer was slightly thinner, but still present, on CLA160 (Fig. 1E, F, G and H, Table 2). The colony morphology of CLA160 was similar to that of CaT2, in which the cell was surrounded by a mycelium-like structure. However, the colony size of CLA160 was slightly smaller than that of CaT2 (data not shown). These results indicated that CLA160 is clearly aggregation-defective and that CaT2 has an unidentified mechanism for cell-to-cell aggregation. Notably, methane production by CLA160 was found to be of the same level and pattern as that by CaT2 under agitation (Fig. 2), suggesting that the mutation does not affect the central metabolism of CLA160.

### Extracellular proteins related to CaT2 aggregation

**Fig. 3:**
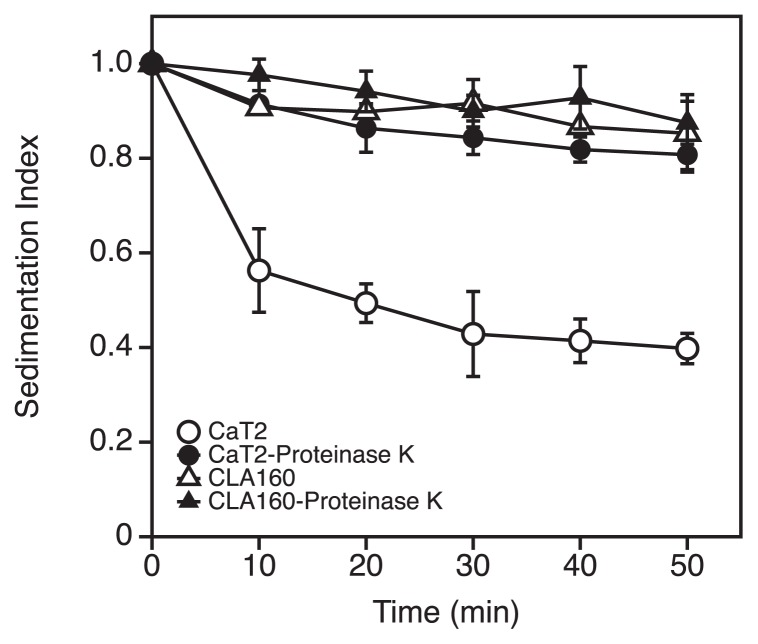
Sedimentation indices of CaT2 and CLA160 and effects of the proteinase K treatment. CaT2 (circles) and the aggregation-defective mutant CLA160 (triangles) were treated with proteinase K (closed) or left untreated (open) and then subjected to a sedimentation analysis. Error bars represent standard deviations of the means of three independent experiments.

Previous studies indicated that biofilm-forming archaea, such as *Methanobrevibacter smithii* and *Sulfolobus solfataricus*, produce an abundant extracellular matrix, mainly composed of polysaccharides and proteins, surrounding their cells (25). To examine whether extracellular proteins are involved in the aggregation of CaT2, a proteinase K treatment experiment was performed, and its effects were evaluated by sedimentation experiments. CaT2 showed a large reduction in the sedimentation index, whereas CLA160 showed almost no difference (Fig. 3), which is consistent with previous findings showing that aggregated cells sediment markedly faster than non-aggregated cells (16). Phase-contrast observations confirmed that the proteinase K treatment caused the dispersion of CaT2 aggregated cells (data not shown). These results suggest that CaT2 has extracellular proteins that are responsible for aggregation, and also that the genes coding for these proteins are defective in CLA160.

### Genomic analysis of the aggregation-defective mutant CLA160

To identify genes encoding for extracellular proteins related to the aggregation of CaT2, CLA160 was subjected to draft genome sequencing followed by direct sequencing. Three single-nucleotide mutations were found in CLA160 (Table 1). Two of them occurred in a non-coding region and the remaining one in the gene *MTCT_1020*, which encodes a hypothetical protein with 3,162 amino acid residues. The mutation in *MTCT_1020* was a single nucleotide substitution that caused a nonsense mutation, resulting in a reduction in the size of the product to 144 amino acid residues. Taken together with the results from the experiments with proteinase K, the product of *MTCT_1020* appears to have lost its function in CLA160 and is responsible for aggregation in CaT2.

### Structure of the hypothetical protein encoded by *MTCT_1020*

**Fig. 4:**
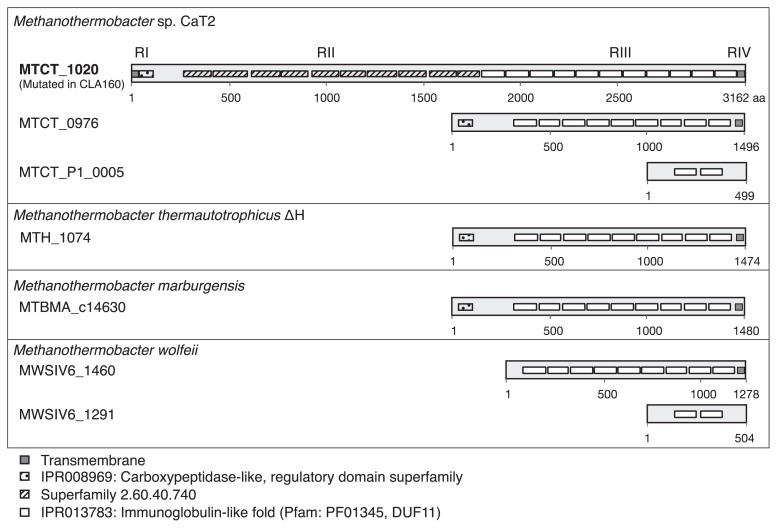
Predicted domain architecture of MTCT_1020 (T2GKU7, the mutated gene of CLA160), MTCT_0976 (T2GJL3), and MTCT_P1_0005 (T2GLL5) (CaT2), MTH_1074 (O27146) (ΔH), MTBMA_c14630 (D9PXU4) (*Methanothermobacter marburgensis*). All datasets were obtained from the UniProt database (http://www.uniprot.org), including InterPro (https://www.ebi.ac.uk/interpro). The numbers depicted under each box are amino acid numbers.

To understand the function of the hypothetical protein encoded by *MTCT_1020*, the protein sequence structure, particularly at the motif and domain levels, was analyzed. Information in the UniProt database showed a signal sequence at its N terminus, a carboxypeptidase-like domain (IPR008969) (RI), a large middle region containing repeated domains separated into two portions (RII: FunFam 3139, CATH Superfamily 2.60.40.740 or RIII: IPR013783 [Pfam: PF01345, DUF11]), and a membrane-spanning sequence at its C terminus (RIV) (Fig. 4). The arrangement of the signal sequence and the single membrane-spanning sequence suggests that the product of *MTCT_1020* is a membrane-bound protein at its C-terminal domain with a large middle region located outside of the membrane. In CLA160, most of the middle domain and the membrane-spanning sequence are absent from the product of *MTCT_1020* due to its nonsense mutation (Table 1). Therefore, it is possible that the large middle region is involved in aggregation in CaT2. The middle region of MTCT_1020 is composed of two different portions: the first repeated region (RII) and second repeated region (RIII) (Fig. 4). RIII contains 11 repeated domains of DUF11 (Pfam: PF01345). However, a domain search analysis using hmmsearch with the hmm model of DUF11 against the UniProtKB database, restricted to *Methanothermobacter* (taxid: 145260) (Table 3) indicated that *Methanothermobacter* species possess proteins, such as MTCT_0976 of CaT2, MTH_1074 of *Methanothermobacter thermautotrophicus* ΔH, and MTBMA_c14630 of *M. marburgensis* (Fig. 4). These proteins are uncharacterized, and in contrast to CaT2, *M. thermautotrophicus* ΔH and *M. marburgensis* do not aggregate (16). Furthermore, another DUF11 domain-containing protein (MTCT_P1_0005) was found in CaT2 by this search, which was not mutated in CLA160 (Fig. 4). These results suggest that the RIII DUF11 domain-repeated region of MTCT_1020 is not important for aggregation, whereas RII, containing conserved repeated domains or the whole structure, may be responsible for the function of MTCT_1020 in the aggregation of CaT2. Notably, the conserved domains in RII (FunFam 3139: CATH Superfamily 2.60.40.740) showed gene ontology (GO) terms such as calcium ion binding (GO:0005509), metal ion binding (GO:0046872), and extrachromosomal circular DNA (eDNA) (GO:0005727), suggesting that other materials, metal ions, and eDNA are involved in the aggregation of CaT2.

**Fig. 5:**
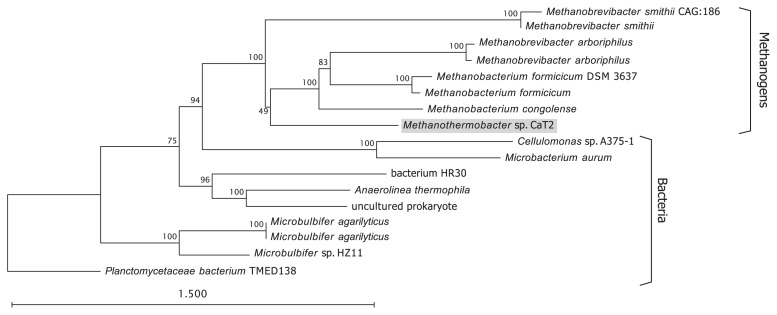
Phylogeny of the *MTCT_1020*-encoded protein and its homologs. A multiple sequence alignment of the amino acid sequences of the *MTCT_1020*-encoded protein and the homologous proteins of *Methanothermobacter* sp. CaT2 (WP_084126234.1: accession number), *Methanobacterium congolense* (WP_084789945.1), *Methanobacterium* sp. MO-MB1 (WP_100905616.1), *Methanobrevibacter arboriphilus* (WP_080459654.1), *Methanobrevibacter arboriphilus* (WP_042702822.1), *Methanobacterium formicicum* DSM 3637 (EKF85388.1), *Methanobacterium formicicum* (WP_082055701.1), *Methanobrevibacter smithii* (WP_019262854.1), bacterium HR30 (GBD25567.1), *Planctomycetaceae bacterium* TMED138 (OUV71746.1), uncultured prokaryote (BAL57189.1), *Anaerolinea thermophila* (WP_013559229.1), *Methanobrevibacter smithii* CAG:186 (CDF29159.1), *Microbulbifer* sp. HZ11 (WP_043320659.1), *Microbulbifer agarilyticus* (AQQ68885.1), *Microbulbifer agarilyticus* (WP_077407055.1), *Microbacterium aurum* (WP_076691662.1), and *Cellulomonas* sp. A375-1 (WP_048344453.1) was generated using ClustalO with default parameters. The phylogenetic tree was subsequently generated using the Create Tree program using the neighbor-joining method (the Kimura correction) and 1,000 bootstrap replications in CLC Sequence Viewer 8.0 (http://www.clcbio.com). The scale bar corresponds to 0.1 substitutions per amino acid.

A DELTA-BLAST search for MTCT_1020 against the non-redundant (nr) database in NCBI indicated that proteins homologous to that encoded by *MTCT_1020* are distributed not only in methanogens, but also in bacteria (Fig. 5). *M. formicicum* and *M. smithii* showed the aggregation phenotype (3, 43) and were previously reported to aggregate in dense granules in methanogenic reactors (43) or microflora (29), respectively. This result supports the contribution of MTCT_1020 to aggregation and suggests an aggregating capability in the listed strains by their MTCT_1020-homologous proteins.

### Effects of DNase I and metal ions on the aggregation of CaT2

**Fig. 6:**
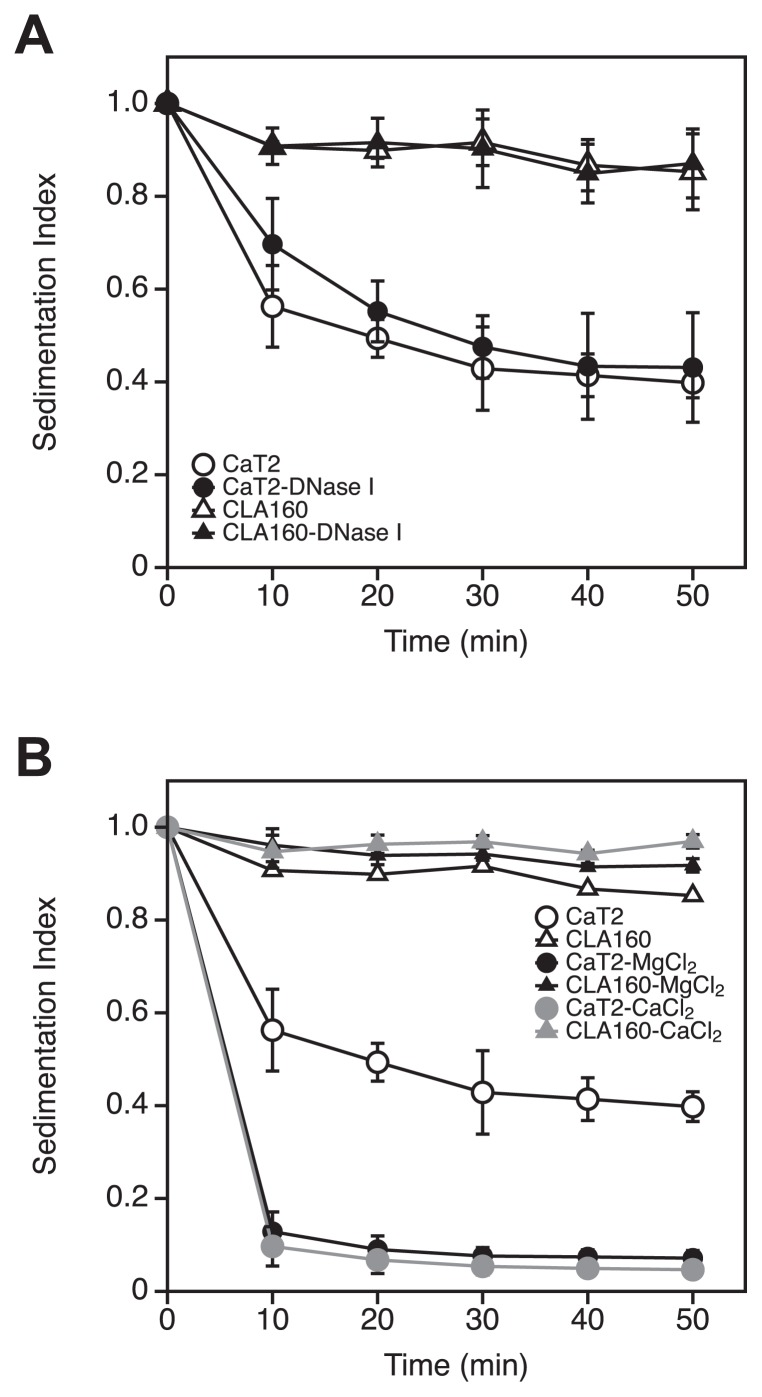
Effects of the DNase treatment or addition of metal ions on sedimentation indices of CaT2 and CLA160. (A) CaT2 (circles) and the aggregation-defective mutant CLA160 (triangles) were treated with DNase I (closed) or untreated (open) and then subjected to a sedimentation analysis. (B) CaT2 (circles) and the aggregation-defective mutant CLA160 (triangles) were treated with CaCl_2_ or MgCl_2_. Error bars represent standard deviations of the means of three independent experiments.

Previous findings suggested that eDNA and metal ions are involved in the aggregation mechanisms of CaT2. eDNA and metal ions have been shown to play a role in the cell-to-cell interactions of microorganisms (25, 31). To elucidate the mechanisms underlying aggregation in more detail, sedimentation experiments on CaT2 and CLA160 were performed following their treatment with DNase I or the addition of the metal ions Ca^2+^ and Mg^2+^. The DNase I treatment had almost no effect on the sedimentation indices of CaT2 and CLA160 (Fig. 6A). Conversely, the addition of CaCl_2_ reduced the sedimentation index of CaT2, but not of CLA160 (Fig. 6B). The addition of MgCl_2_ had a similar effect to that of Ca^2+^ ions on CaT2 (Fig. 6B). These results suggest that Ca^2+^ or Mg^2+^ ions enhance the aggregation of CaT2.

## Discussion

In the present study, we isolated the aggregation-defective mutant, CLA160, from *Methanothermobacter* sp. CaT2, which normally forms aggregated clumps via tight cell-to-cell interactions (Fig. 1). Although it has been proposed that the cell-to-cell interactions of CaT2 are mediated by a surface sugar layer (16), TEM observations suggest that CLA160 retains a surface sugar layer that is thinner than that of CaT2 (Table 2). Seven genes have been predicted to be responsible for aggregation in CaT2 (16); however, there were no mutations in these genes in CLA160, and the remaining mutations in CLA160 were located in the non-coding regions of its genome (Table 1). The proteinase K treatment and a genomic analysis of CLA160 showed that *MTCT_1020*, which encodes a putative extracellularly protruding membrane protein, plays a role in the dense aggregation properties of CaT2. Since protein-mediated cell-to-cell adhesion may be observed in many microorganisms (2, 4, 6, 23), protein-mediated aggregation is not unexpected. We presume that MTCT_1020 is a protein that contributes not only to the aggregation, but also to the stabilization of the surface sugar layer. However, a low level of aggregation was observed morphologically in CLA160 (Fig. 1B), while no obvious proteinase K effect was observed in this strain (Fig. 3), suggesting that another mechanism, other than a protein-mediated one, is involved in the aggregation of CaT2. One possibility is the sugar layer, which remains present in CLA160 (Fig. 1H).

The protein encoded by *MTCT_1020* showed a specific sequence structure, with its signal sequence and single membrane-spanning sequence suggesting that this protein is membrane-anchored at its C-terminal domain and its RII is important for cell-to-cell interactions (Fig. 4). This RII portion may be related to adhesion and metal ion binding, which is supported by a Conserved Domain Search (https://www.ncbi.nlm.nih.gov/Structure/cdd/wrpsb.cgi), identifying it as a D2 domain. This D2 domain consists of the repetition of the major component RrgB, which is also the main component that forms the backbone in *Pneumococcal* pili (36). Therefore, these regions may bind with each other. Although our prediction suggests that RIII containing the repeated DUF11 domain is not responsible for aggregation, DUF11 is often found in membrane proteins in a small number of phylogenetically distant prokaryotes. In the *MTCT_1020*-encoded protein, each of the DUF11 repeat sequences overlapped with sequences of five to 10 motifs of 11 DUF11 domains consisting of 120-residue repeats. DUF11-containing repeat domains are not associated with aggregation, but may be important for stabilizing the surface cell wall structures of CaT2.

Ca^2+^ or Mg^2+^ ions enhance the aggregation of CaT2, but not that of CLA160 (Fig. 6B). This result prompted us to speculate that metal ions stimulate cell-to-cell interactions via a direct association with the *MTCT_1020*-encoded protein. The enhancement of cell flocculation by metal ions has been reported for several microorganisms (28, 35, 41). In the halo-tolerant archaea *Halobacterium salinarum*, Ca^2+^ ions were found to be essential for initiating cell aggregation, while Mg^2+^ ions did not induce the flocculation of cells (13). The pathogenic, Gram-negative, auto-aggregating bacterium *Legionella pneumophila* exerted a metal ion enhancement effect on an auto-aggregation process, which is mediated by the *Legionella* collagen-like protein in a divalent cation-dependent manner (1). Moreover, in the eukaryote *Saccharomyces cerevisiae*, Ca^2+^-dependent, lectin-like interactions were found between proteins related to flocculation and specific sugar residues on the surfaces of other cells (14, 40). Therefore, aggregation enhancement mechanisms by proteins and cationic ions are common and widespread among microorganisms. Interestingly, the enhancement of start-up granulation by the addition of Ca^2+^ ions has also been reported in UASB reactors (5, 26, 37). The functions of proteins homologous to that encoded by *MTCT_1020* are mostly unknown (Fig. 5). An investigation into the relationships between MTCT_1020 and metal ions and their influence on aggregation may enhance current knowledge on the aggregating mechanisms of methanogens and may also supply new information about other aggregating microorganisms. Finally, our results suggest that an extracellular protein that possesses repeated sequences and a unique structure is a core factor in the aggregation of *Methanothermobacter* sp. CaT2.

## Figures and Tables

**Table 1:** Summary of mutations in CLA160.

Position	Gene	Product	Mutation	Amino acid substitution	Amino acid number
1022836	*MTCT_1020*	Hypothetical protein	Nonsense mutation C→A	Gly166Stop	144
1180340	Non-coding	—	Deletion T	—	—
1614615	Non-coding	—	Nucleotide substitution G→T	—	—

**Table 2:** Thickness of the cell wall (CW) and sugar layer (SL) in methanogens.

Strain	CW^a^	SL^a^
CLA160	15.6±1.3	8.8±0.63
CaT2	15.2±1.3	12.7±0.8

a The thicknesses of layer structures were measured for 20 different cells in several TEM images. The value of thickness is expressed as a mean ± standard deviation.

**Table 3:** The hmmsearch using the DUF17 model against *Methanothermobacter* protein sequences in the UniProt database.

Strain	Aggregation^a^	UniProt No.	Protein	Locus_tag	Length	E-value^b^
*Methanothermobacter* sp. CaT2	+	T2GKU7	Uncharacterized protein	MTCT_1020	3,162	1.8E-217
		T2GJL3	Uncharacterized protein	MTCT_0976	1,496	4.1E-182
	T2GLL5	Uncharacterized protein	MTCT_P1_0005	499	1.6E-18	
*Methanothermobacter thermautotrophicus* ΔH	–	O27146	Putative membrane protein	MTH_1074	1,474	2.4E-188
*Methanothermobacter marburgensis* Marburg	–	D9PXU4	Uncharacterized protein	MTBMA_c14630	1,480	3.3E-177
*Methanothermobacter wolfeii*	ND	A0A1M4MTN5	Uncharacterized protein	MWSIV6_1460	1,278	5.7E-163
		A0A1M4MT64	Uncharacterized protein	MWSIV6_1291	504	5.8E-17
*Methanothermobacter* sp. EMTCatA1	ND	A0A223YY22	Uncharacterized protein	tca_01038	1,475	2.1E-180

a ND, no data.

b The search threshold was <1E-10.
